# A Coarse-Grained Force Field for Silica–Polybutadiene Interfaces and Nanocomposites

**DOI:** 10.3390/polym12071484

**Published:** 2020-07-02

**Authors:** Alessio David, Marta Pasquini, Ugo Tartaglino, Guido Raos

**Affiliations:** 1 Department of Chemistry, Materials and Chemical Engineering, “G. Natta”, Politecnico di Milano, 20131 Milan, Italy; alessio.david@polimi.it (A.D.); marta.pasquini13@gmail.com (M.P.); 2 Pirelli Tyre S.p.A., 20126 Milan, Italy; ugo.tartaglino@pirelli.com

**Keywords:** silica, rubber, molecular dynamics, coarse-graining, payne effect

## Abstract

We present a coarse-grained force field for modelling silica–polybutadiene interfaces and nanocomposites. The polymer, poly(cis-1,4-butadiene), is treated with a previously published united-atom model. Silica is treated as a rigid body, using one Si-centered superatom for each SiO${}_{2}$ unit. The parameters for the cross-interaction between silica and the polymer are derived by Boltzmann inversion of the density oscillations at model interfaces, obtained from atomistic simulations of silica surfaces containing both $Q^{4}$ (hydrophobic) and $Q^{3}$ (silanol-containing, hydrophilic) silicon atoms. The performance of the model is tested in both equilibrium and non-equilibrium molecular dynamics simulations. We expect the present model to be useful for future large-scale simulations of rubber–silica nanocomposites.

## 1. Introduction

Polymer nanocomposites (PNCs) are obtained by dispersion of different types of nanoparticles (NPs) within a polymer matrix. They have been the subject of intensive research over the last couple of decades, after it was discovered that the use of nano-sized fillers (particles, tubes or platelets, depending on their aspect ratios) could yield dramatic changes to the polymer properties [1,2,3]. Substantial improvements to the mechanical, optical, electrical or other properties could be achieved at relatively low particle volume fractions, much smaller than would have been expected on the basis of the established models for conventional composites with micron-sized particles (e.g., <5% versus >20%, say). Within a few years came the realization that one key factor for this enhancement was the presence of an interfacial region of thickness δ surrounding the particles, in which the matrix properties deviate appreciably from those in the bulk [4,5]. Since δ can be of the order of tens of nanometers [6], its presence makes a big difference for PNCs. In fact, these might be described as three–phase materials (particle, bulk matrix and interfacial matrix), as opposed to the two-phase description which applies to conventional composites [7].

Filled rubbers are a special class of PNCs, in which the matrix is a cross-linked elastomer and the NPs (“fillers”) can be carbon black, silica, or a combination of the two. They have been known and exploited for a long time in automotive tires and other rubber goods, well before the “nano” keyword came into fashion [8]. The mechanical reinforcement which occurs in these system is a complex phenomenon. From a phenomenological point of view, it involves a strong increase in the small-strain modulus of the material, a non-linear drop in the modulus at moderate deformations (Payne or Mullins effect), a marked improvement in the resistance to rupture and abrasion [9,10]. Empirical laws and mastery in compounding are still key to the application of these materials, but in recent years the problem has been attacked also by physics-based theories and models [11]. Traditionally, rubber reinforcement was associated with the presence of “secondary network” formed by physical association of the filler NPs [12,13]. Later on, it was suggested that the conformation and dynamics of the polymer chains within the “glassy shell” or “bound rubber” layer surrounding the particles is critical for reinforcement [14,15,16]. This shell is closely related to the interfacial layer mentioned in previous paragraph. The two competing descriptions, emphasizing particle–particle and polymer-particle interactions, are in fact complementary, as the interparticle contacts making up the filler network are likely to be polymer-mediated, rather than being “naked”.

The use of computer simulations to explore the behaviour of PNCs and rubber reinforcement is a relatively new but quickly expanding area of research [17]. Thanks to the continuous improvements in computer hardware and molecular dynamics (MD) software, atomistic or all-atom (AA) simulations have become possible also in this field, with silica [18,19,20,21,22,23,24,25,26] and sp^2^ carbon structures [27,28,29,30,31] as popular NPs. In principle, AA models would allow a direct comparison with experimental results. However, their use poses a number of significant challenges. First of all, the high sensitivity of interfacial properties to the parameters used in simulations requires the use of accurate force fields (FFs). As extensively reviewed by [32], building a classical FF over electronic structure calculations is not an easy task, especially for polymers, where there is an interplay between enthalpic and nonlocal entropic contribution. The interface between organic and inorganic materials is especially difficult, as the FFs which can be used to model them individually were initially developed by different communities and for different purposes. Secondly, dealing with very large systems (>$10^{5}$ atoms) over long times is almost mandatory, in order to describe situations with nanoscale heterogeneities. Thus, the computational cost of AA simulations can quickly become prohibitive. Finally, experimental knowledge about the molecular-level structure of the systems is usually incomplete, so that the simulations invariably rely on some reasonable but untested assumptions.

Since AA simulations impose limits on system dimension, most of them focus on the structure and dynamics at the interface between the polymer and one or two particles [18,19]. One notable exception is represented by the simulations performed by Pavlov and Khalatur [21], with 590,000 atoms divided among 80-unit poly(cis-1,4-butadiene) (PB) chains and 64 silica NPs. These system sizes are impressive by current standards, but they still fall short of those required to model the hierarchical organization of the nanoparticles into fractal-like structures, such as those which characterize many rubber nanocomposites [33]. Thus, most simulations of PNCs are still carried out with coarse-grained (CG) models, typically based on bead-and-spring polymer chains and Lennard–Jones-type (LJ) interaction potentials [34,35,36,37,38,39] or some appropriate generalization of them [40]. An even coarser description is the one based on Dissipative Particle Dynamics (DPD) [41,42]. A CG model simplifies the treatment and typically allows a ten-fold increase in both the system size and the simulated time. One important advantage of these CG models is the absence of long-range electrostatic interactions, whose evaluation uses up a very significant fraction of the CPU time in AA simulations. There are situations where electrostatic interactions are essential also within a a CG model [43,44], for example when dealing with polyelectrolyte networks [45], but this does not apply to a typical hydrocarbon-based elastomer. Of course, the computational gains which come from coarse-graining are usually accompanied by some loss in chemical specificity [46].

The aim of this work is to develop and test a model of silica-rubber systems which retains as much as possible the chemical detail in the description of these materials, but introduces some key simplifications which will allow large-scale simulation of their nanocomposites, similar to CG models. Thus, the present study represents the first step within a broader, longer-term research project. For the polymer, we restrict our investigation to poly(cis-1,4-butadiene) [47,48]. The choice of this particular combination of materials is determined by its widespread use in the tyre industry, combined with its relative simplicity in terms of structure and chemical composition. Following previous experimental and computational approaches [6,49,50,51,52,53], we study the behaviour of the polymer at the interface with the filler by sandwiching it between two parallel surfaces. In addition to developing the model, we study its non-equilibrium behaviour. Specifically, we have performed a computational Dynamical Mechanical Analysis (DMA) to investigate the critical issue of the Payne effect.

The paper is organized as follows: in Section 2 we present the models used for the polymer and for silica. In Section 3 we briefly describe the methodologies adopted for the equilibration of the systems, the method used for the silica parametrisation and, lastly, the DMA. The first part of the discussion in Section 4 focuses on the transferability of the parametrisation to other temperatures. In the second part of the section we comment the DMA results. We summarize our conclusions in Section 5.

## 2. Models

We represent PB by a well-established united-atom model [54,55], which suppresses hydrogen atoms by utilising carbon superatoms that implicitly take them into account. This coarser representation results in slimmer simulations, while preserving important molecular details such as rotational barriers. Compared to the AA representation, the speedup arises from the smaller number of atoms and the exclusion of electrostatic interactions, since the (small) charges which would be present on the C and H atoms exactly compensate each other. Such an approximation is reasonable for hydrocarbons, as they are prototypical apolar compounds. In addition, the potential energy surfaces describing intra- and inter-molecular interactions tend to be smoother for CG model, and this results in faster dynamics in comparison with AA ones [56,57,58].

The surface of silica is characterised by complex and variegate structures, depending on its preparation conditions and post-preparation treatments [59]. Silicon atoms at silica surfaces complete their valency with hydroxyl groups (silanols). Following the NMR literature, they are classified as $Q^{1}$, $Q^{2}$, $Q^{3}$, $Q^{4}$, depending on the number of bulk O atoms bonded to them (i.e., $Q^{4}$ corresponds to bulk SiO${}_{2}$ without silanols). Nanoparticle size influences the silanol coverage, and thermal treatment lowers the surface concentration of silanol groups by water elimination. A recent publication describes a database of silica surfaces and the associated AA force field [60], which can be used to model such surfaces. We have adopted it as the starting point for the development of our CG silica model. Consistently with the united-atom description of the polymer, we employ one superatom per SiO${}_{2}$ unit. This simplification should preserve the essential ingredients of the problem, allowing the simulation of the salient features of rubber reinforcement by silica. A similar CG silica model was developed by Müller-Plathe and coworkers [61,62], but it was designed from the outset to be used with a specific CG polystyrene model.

**Figure 1:**
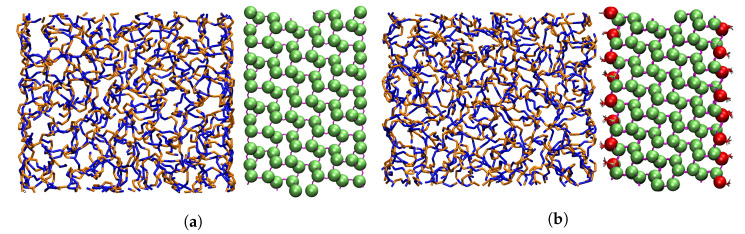
(**a**) Superposition of the AA and CG $Q^{4}$ silica wall in front of the polymer. AA silica is represented by lines partially covered by CG $Q^{4}$ beads represented as green spheres. (**b**) Superposition of the AA and CG $Q^{3}$ silica surface in front of the polymer. CG $Q^{4}$ beads are represented as green spheres and CG $Q^{3}$ beads as red spheres.

We introduce and derive the potentials for two types of silica superatoms. This will allow us to model silica NP’s with different chemistries [59,60], including the possibility of local heterogeneities which are known to affect the polymer dynamics [63,64,65]. One model has siloxide bridges without silanol groups, so that all silicon atoms can be classified as $Q^{4}$. The other surface has 4.7 silanol/nm${}^{2}$ at the surface. The silicon atoms bonding to one silanol are classified as $Q^{3}$, while the remainder are still $Q^{4}$. We will use the same shorthand notation also for the surfaces, referring to the one without silanol groups as $Q^{4}$ and the other one as $Q^{3}$ (Figure 1). The $Q^{3}$ surface from the database [60] was obtained by cleaving α-cristobalite, a quartz polymorph, along 20$\bar{2}$ plane. According to the authors, this best represents the surface structure for particles smaller than 200 nm. The $Q^{4}$ surface was obtained by complete condensation of surface silanol groups, followed by energy minimization. The resulting AA structures are then coarse-grained by deleting all oxygens and hydrogens and centering one superatom on each silicon.

In all simulation, the silica structure is kept rigid, since it has elastic constants that that are orders of magnitude higher than the polymer matrix. The non-bonded interactions involving the silica superatoms have the LJ functional form, with parameters obtained by Boltzmann inversion. There are no charges in the CG silica model, unlike the AA one. The neglect of electrostatic charges does not affect the silica–pB interactions, since there are no charges on the polymer. On the other hand, it is clear that this model could not be used to model accurately the interaction between bare silica NPs. This does not represent a major limitation for the model, because these direct interactions are unlikely to occur in practice. The reason is that silica NPs are usually grafted with non-polar coupling agents that bind covalently to the polymer chains, to improve their dispersion and promote adhesion at the rubber–filler interface. These coupling agents will not be explicitly simulated here, but they will be introduced in a future publication, dealing also with cross-linked PB matrices.

## 3. Methods

### 3.1. Equilibration

For the force field parametrisation we built two fully periodic system containing one silica slab, with either $Q^{3}$ or $Q^{4}$ surfaces on both sides, sandwiching a melt of 200 PB 10-mers. The periodic cell is 67 Å × 70 Å in the xy plane and the silica slab is 21 Å thick in *z* direction. The number of PB chains ensures that the thickness of the polymer layer is about 45 Å after equilibration, enough to observe the damping of density fluctuations and a bulk-like behaviour at its center. Although there is only one silica wall in the simulation box, the periodicity in the *z*-direction guarantees that the polymer is effectively confined between the upper surface of one slab and the lower surface of its periodic image.

The initial configuration of the AA systems was generated by randomly replicating and rotating a sample chain within the space not occupied by the silica wall. The initial length of the box in the *z* direction was chosen to achieve an average density of 0.93 g/cm${}^{3}$ within the polymer, representing a common value for many elastomers [66]. The first part of the equilibration was performed at 500 K, to guarantee high mobility of the chains. In the initial stages, we used a so-called “soft potential” in the form:

(1) $$V_{ij}^{\mathrm{soft}}\left(r\right)=A\left[1+\cos\left(\frac{\pi r}{\sigma_{ij}}\right)\right]$$

which accounts only for the repulsive part and it is truncated at $r=\sigma_{ij}$. During an NVT run, the prefactor *A* grows from 0 to 20 kcal/mol, allowing the polymer beads to get out of each other’s way and the chains to loose memory of their initial conformational states, as monitored by the relaxation of the end-to-end vector. At the end of this step, the polymer beads are evenly dispersed and it is possible to switch to the steeper LJ potential:

(2) $$V_{ij}^{\mathrm{LJ}}\left(r\right)=4\epsilon_{ij}\left[{\left(\frac{\sigma_{ij}}{r}\right)}^{12}-{\left(\frac{\sigma_{ij}}{r}\right)}^{6}\right].$$

After reintroducing the LJ potential, the energy of the system is minimised to eliminate any residual bead overlaps and a 2 ns NPT simulation is run to converge the density and the end-to-end distance of the chains. The pressure is adjusted to 1.0 atm by an anisotropic NPT simulation, in which the system’s periodicity in the *Z* direction (i.e., perpendicularly to the silica surfaces) is allowed to change in order to control the density of the confined polymer.

All the simulations have been carried out using LAMMPS (Large-scale Atomic/Molecular Massively Parallel Simulator) [67,68]. The cutoff for the evaluation of the LJ interactions was 12 Å. The integration timestep was 2 fs.

### 3.2. Boltzmann Inversion

After the equilibration steps, the system is cooled down to 298 K by an NPT simulation and the density oscillations of the polymer in front of the silica wall are registered. The structure of the surface is then coarse-grained by deleting non-silicon atoms. At this point, the full Boltzmann inversion procedure would involve a numerical interpolation of the CG potentials [69]. Here instead we adopt a simplified version, in which the CG potentials retain the LJ form. The LJ parameters are tuned to match the density oscillations obtained during the AA simulation. The LJ cutoff and the MD timestep for these CG simulations are identical to those of the AA ones.

Our silica model accounts for two types of Si superatoms or beads, to match the chemical landscape of the reference AA walls. There are a $Q^{4}$ bulk bead that does not bond to any OH group and a $Q^{3}$ surface bead that is bonded to one OH group. The $Q^{4}$ superatoms are parametrised first, by applying the Boltzmann inversion to the $Q^{4}$ surface. Then the $Q^{3}$ surface is parametrised by using the previously parametrised $Q^{4}$ beads within the bulk, while the parameters of the $Q^{3}$ superatoms at the surface are tuned to match the density oscillations of the polymer in front of the AA silica wall. Only the diagonal LJ parameters for the superatoms are tuned, while the parameters for the cross interactions are obtained by the geometric mixing rules ($\epsilon_{ij}=\sqrt{\epsilon_{i}\epsilon_{j}}$ and $\sigma_{ij}=\sqrt{\sigma_{i}\sigma_{j}}$) [70,71].

### 3.3. Dynamical Mechanical Analysis

**Figure 2:**
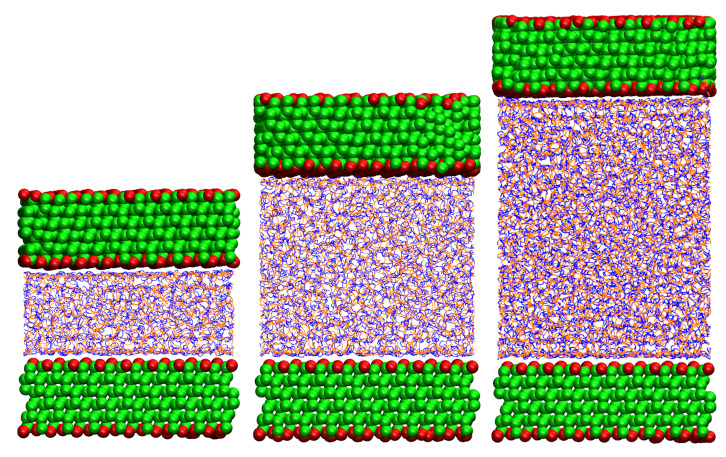
From left to right: systems $l_{1}$, $l_{2}$, $l_{3}$.

For the DMA, we built three sandwich systems composed of two $Q^{3}$ silica walls intercalated by a layer of PB chains. The system is xy periodic, but it is finite in the *z* direction. The chain length in this set of simulations was increased from 10 to 100 monomers, to study the effect of chains bridging the surfaces. For a given chain length, bridging decreases and then vanishes on increasing the wall-to-wall separation. This simulation setup is similar to that in experimental studies of confined polymer films using the surface force apparatus [72,73] and atomic force microscopy [74], which have shown major changes in the film behavior due to polymer bridging. The surface separation can also be roughly interpreted as an average inter-NP distance within a nanocomposite. The Payne effect is known to be amplified by increasing the concentration of NPs or decreasing their dispersion [12]. We tested three systems with different separations, in order to highlight the threshold for the occurrence of the effect. The distances are chosen to represent three scenarios in which the totality, a fraction, or none of the chains bridge the walls. After the equilibration, the distances between the walls are $l_{1}$ = 27.7 Å, $l_{2}$ = 56.6 Å, $l_{3}$ = 90.0 Å, shown in Figure 2.

The equilibration of the systems is performed in a similar fashion to the one described before, but skipping the atomistic simulation of the walls. The production run DMA is performed at 250 K, lower than room temperature but above the glass transition temperature of PB, to reduce the thermal fluctuations of the stress that partly mask the signal. The shear in the polymer layer is induced by forcing one surface to move sinusoidally and parallel to the layer, whilst the other surface remains static. In the approximation of laminar flow, the induced shear deformation inside the polymeric layer is:

(3) $$\gamma_{=}\gamma_{0}\sin\left(\omega t\right),$$

where $\gamma_{0}$ is the shear amplitude (defined below), ω is the angular frequency (0.1 GHz) of the imposed deformation and *t* is the time variable. The $\gamma_{0}$ range considered is different for every system and it has been chosen to include $\gamma_{0}$ = 1%, where the Payne effect occurs experimentally. The deformation frequency is much higher than those that are commonly employed in experimental studies of filled rubbers, but not so high to produce a glassy response of the polymer (see the results section below). It is in line with those employed in other, current simulation studies of polymer networks and nanocomposites [37,75].

The shear component of the stress inside the polymeric layer $\sigma_{\alpha \beta }$ is recorded and analyzed. It has an oscillatory behaviour as well, but it is phase-shifted due to viscous phenomena. The heat produced during the deformation cycles must be extracted, in order to keep the system’s temperature stable. We have employed a Nose-Hoover thermostat with 0.2 ps relaxation time, identical to that for the equilibrium simulations. This is far shorter than the oscillation period (10 ns), therefore it is adequate to keep the temperature constant.

In most simulations, the first atomic layers of the polymer were forced to move following the rigid surface next to them. This “stick” boundary condition provides a simplified description of a perfectly bonded polymer–filler system. The thickness of the layer is extracted from the density curves, as the distance at which the first minimum occurs. One system, denoted as $l_{1}^{*}$, has a inter–surface distance equal to $l_{1}$, but the chains are free to move throughout the polymer layer. This allows to verify the effect of slippage at the surfaces. Consequently, $\gamma_{0}$ in Equation (3) is calculated as ratio between the maximum displacement of the upper surface and the distance between the two minima defining the adsorbed polymeric layers. In system $l_{1}^{*}$, the distance considered is that between the centers of the silica atoms belonging to the two surface layers. The stress $\sigma_{\alpha \beta }$ is similarly calculated by summing the per-atom stresses within the mobile part of the polymeric layer, and dividing by its volume [76]. The number of complete cycles performed for systems $l_{1}$, $l_{1}^{*}$, $l_{2}$ and $l_{3}$ are 6, 6, 18 and 9, respectively.

## 4. Results

### 4.1. Force Field Parameters and Transferability

**Figure 3:**
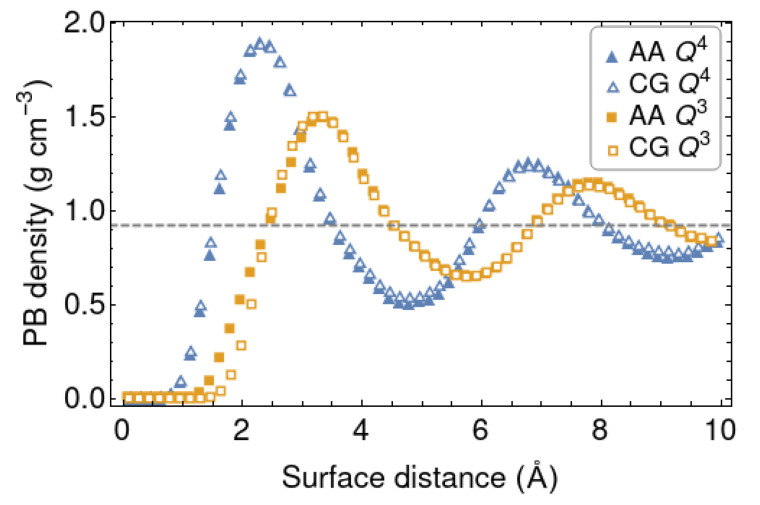
Density profiles of the polymer in front of the AA and CG silica wall models at 298 K, for two silica surface models. The average density at the center of the slab is shown with a dashed line.

In this part we discuss the AA and CG simulations with the shorter PB chains (10-mers). The LJ parameters coming from the Boltzmann inversion at 298 K are collected in Table 1, together with those of the polymer [54,55]. The density distribution of polymer beads are compared in Figure 3. Overall, the match between the density curves of the polymer in front of the CG and AA silica walls is rather good. It is fully satisfactory for the $Q^{4}$ surface, slightly less for the $Q^{3}$ one. The individual distributions of CH ($sp^{2}$) and CH${}_{2}$ ($sp^{3}$) beads follow a similar behaviour. The positions and heights of the peaks mainly reflect the values of σ and ϵ for the cross LJ potentials, respectively. The net interaction between the polymer and silica is stronger for the $Q^{4}$ surface, which has a smoother topography. The silanol groups sticking out of the $Q^{3}$ surface endow it with a more irregular topography and prevent the efficient packing of the polymer chains next to it. This is reflected in the CG model by an increased σ and a lower ϵ.

In order to test the reliability and transferability of our parametrisation, we investigated the effect of coarse-graining on different properties of the confined polymer melt. We repeated the simulations at different temperatures (250 K, 273 K and 325 K) to evaluate the transferability of the force field to different thermodynamic states. The model parameters obtained at 298 K were used also at the other temperatures, without any further adjustments. Of course, this transferability is necessarily approximate, as any coarse-grained potential is effectively an interaction free energy with a temperature-dependent entropic contribution. The change in temperature was accompanied by a change in surface distance, to relax the pressure to 1 atm. At all the considered temperatures, the polymer density profiles obtained with the AA and CG silica walls are in good agreement, as the height and position of the peaks are very well reproduced. Since the density profiles are essentially overlapping, they are not reported.

The gyration tensor is an equilibrium property that describes the perturbation in the overall polymer conformations produced by the confining walls. For a chain of *N* atoms it is calculated as:

(4) $$\langle S_{mn}^{2}\rangle =\langle \frac{1}{N}\sum_{i=1}^{N}\left(r_{m}^{\left(i\right)}-r_{m}^{\mathrm{CM}}\right)\left(r_{n}^{\left(i\right)}-r_{m}^{\mathrm{CM}}\right)\rangle$$

where $r_{m}^{\left(i\right)}$ and $r_{m}^{\mathrm{CM}}$ are the Cartesian coordinates of the *i*th atom and of the chain’s center-of-mass, respectively. The brackets indicate an average over all chains and over time. For symmetry reasons, we may average the components parallel to the walls (xx and yy) and denote the result as $\langle S_{\left|\right|}^{2}\rangle$. The overall radius of gyration us thus $\langle S^{2}\rangle =2\langle S_{\left|\right|}^{2}\rangle +\langle S_{zz}^{2}\rangle$.

**Figure 4:**
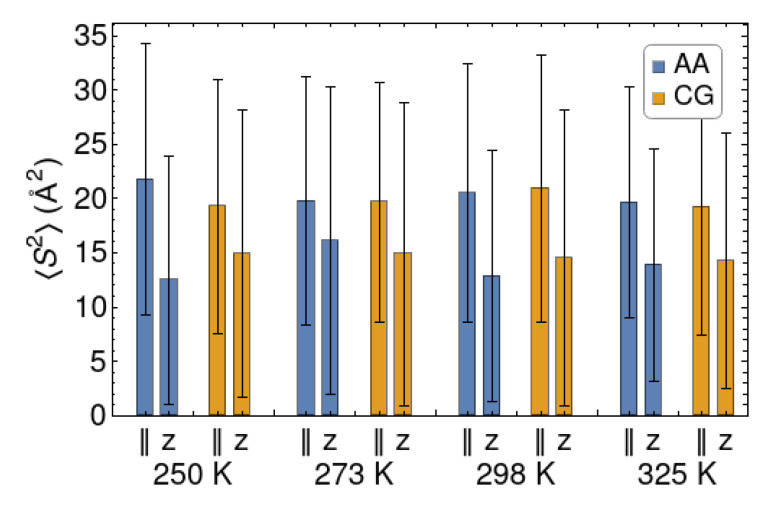
Gyration tensor components for the $Q^{4}$ surface at different temperatures, for the AA and CG models. The zz component is indicated as *z*, while the averaged xx, yy component is indicated with the “||” symbol (parallel, planar). The “error bars” indicate the standard deviations of the distributions.

The parallel and perpendicular components of the radius of gyration are well reproduced at all the considered temperatures (Figure 4). The parallel components of the gyration tensor are consistently larger than their perpendicular counterpart. On average the chains have an oblate shape, as a result of their interaction with one or the other wall. We point out that they are not “squeezed” by the walls, as their $\langle S_{zz}^{2}\rangle$ values are much smaller than the square of the wall-to-walls distance (45 Å). The chains are not long enough to form bridges between the surfaces. In fact, the chains residing at the center of the polymer slab tend to be nearly spherical, while some others are completely adsorbed on one wall throughout a simulation, so that their perpendicular component is close to zero. This variability is responsible for the large “error bars” in Figure 4, which indicate the variance of the distribution of the radii of gyration. Thus, although the distribution of chain sizes is quite broad, both the AA and CG models indicate a flattening of the chains upon confinement.

To test the dynamical properties of the polymer chains, we computed the mean-square displacement (MSD) in the direction orthogonal to the silica walls and extracted the polymer diffusion coefficient $D_{z}$, by fitting it according to the linear relationship:

(5) $$MSD_{z}\left(t\right)=C+2D_{z}t.$$

**Figure 5:**
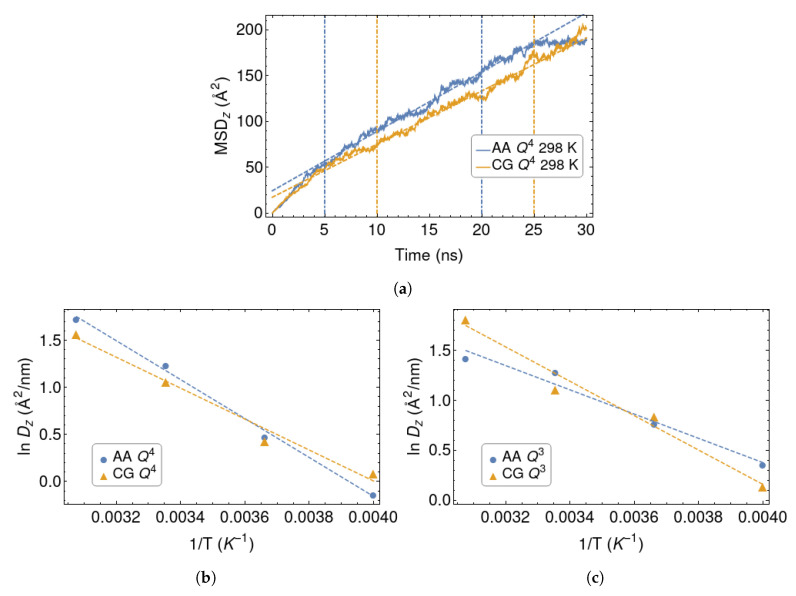
(**a**): Mean-square displacement in the *z* direction for the CG and AA $Q^{4}$ silica surface. Vertical dashed lines delimit the range considered in the linear fits. (**b**,**c**) Diffusivities of the polymer chains in the orthogonal direction, for the $Q^{4}$ and $Q^{3}$ silica walls. Arrhenius fits are shown as dashed lines.

Here *C* is a constant allowing a non-zero intercept at t=0, which we associate with a sub-diffusive behavior at short times. Two examples of such fits are shown in Figure 5a. Note that, because of the confining effect of the surfaces, $MSD_{z}\left(t\right)$ cannot grown indefinitely and must saturate at a value of the order of the squared wall-to-wall distance, when t→∞. This seems indeed to occur, at least in one of the examples. In any case, it is easy to identify a wide range of times where the MSD closely follows Equation (5), allowing a reliable evaluation of the diffusion coefficient in all cases.

The values of $D_{z}$ at different temperatures are plotted in Figure 5b,c. They have been plotted in the Arrhenius form, in order to extract the activation energies $E_{a}$:

(6) $$\ln D_{z}=\ln D_{0}-\frac{E_{a}}{RT}$$

where *R* is the gas constant. Their values are collected in Table 2.

It is interesting, albeit somewhat disappointing, that the coarse-graining procedure induces opposite trends in $E_{a}$ for the two models of silica. For the $Q^{4}$ surface, the coarse-graining lowers the activation energy, while it has the opposite effect in the $Q^{3}$ case. The two AA systems have remarkably different activation energies, with system $Q^{4}$ characterised by higher value. Instead, $E_{a}$ is similar for the two CG surface models. As we had anticipated, some chemical specificity is lost as a result of the coarse-graining process. One should also bear in mind that these diffusion coefficients average the behaviour of chains which are close or far away from the surfaces, resulting in a further loss of information.

The diffusion coefficient represents a chain-level descriptor of the polymer dynamics. More local, segment-level dynamical properties can also be explored. The adsorption/desorption kinetics of individual monomers interacting with the silica surfaces are especially interesting. For this purpose, we introduce the variable $S_{i}\left(t\right)$, which takes the value 1 or 0 if monomer *i* is adsorbed or desorbed at time *t*. A monomer is considered ‘adsorbed’ if its distance from the surface is less than the distance at which the density profile reaches the first minimum (different for the two surfaces, as shown in Figure 3). The overall desorption function is then defined as:

(7) $$\phi_{\mathrm{des}}\left(t\right)=\frac{\sum_{i}\left[S_{i}\left(t\right)-\langle S\rangle \right]}{\sum_{i}\left[S_{i}\left(0\right)-\langle S\rangle \right]}=\frac{\sum_{i}\left[S_{i}\left(t\right)-\langle S\rangle \right]}{\sum_{i}\left[1-\langle S\rangle \right]}$$

where the summations run over all the monomers initially adsorbed onto the surface.

**Figure 6:**
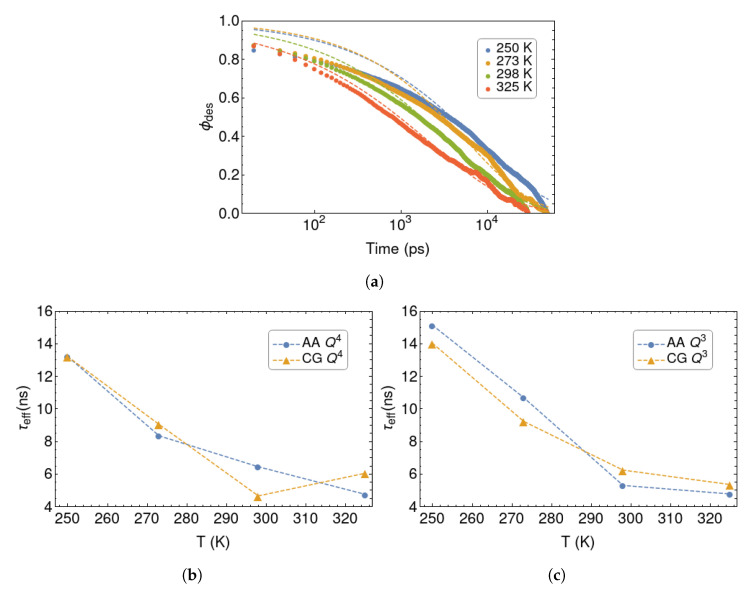
(**a**) $\phi_{\mathrm{des}}$ for PB in contact with an AA $Q^{4}$ silica wall, at different temperatures. The fit to the stretched exponential function is reported with a dashed line. (**b**) *τ*_eff_ as a function of temperature for AA and CG $Q^{4}$ silica wall, and AA and CG $Q^{3}$ silica wall (**c**).

Figure 6a shows the $\phi_{\mathrm{des}}\left(t\right)$ functions as obtained from simulations with the polymer melt in contact with an AA $Q^{4}$ silica wall. The curves obtained with the other silica models have similar trends. They can all be fitted with a stretched exponential function:

(8) $$\phi_{\mathrm{des}}\left(t\right)=e^{-{\left(t/\tau \right)}^{\beta }}$$

where τ is the relaxation time and β is the stretching parameter. A value of β significantly less than unity indicates a dynamically heterogeneous situation, with a coexistence of fast- and slow-desorbing monomers. In such cases, an effective desorption time can be calculated as:

(9) $$\tau_{\mathrm{eff}}=\int_{0}^{\infty }\phi_{\mathrm{des}}\left(t\right)dt=\frac{\tau }{\beta }\Gamma [\frac{1}{\beta }]$$

where Γ is the Gamma function. The values of $\tau_{\mathrm{eff}}$ are plotted in Figure 6b,c, while the individual fit parameters are collected in Table 3. The overall quality of the fits is satisfactory. Note that the fit is dominated by the long-time behaviour, because there are many more points in this range. The short-time decay (t<1 ns) depends on fast local motions that could have been described by a separate functional form. Again, the general trends in the desorption times are respected, but we observe some loss of chemical specificity on going from the AA to the CG model.

### 4.2. Dynamical Mechanical Analysis

DMA is an experimental technique in which a sample of material is deformed in a cyclic fashion. As explained in the Methods section, here we have applied a shear deformation to the confined polymer layers by moving one surface horizontally, while keeping the other fixed. From the stress response it is possible to extract the effective $G^{\prime }$ and $G^{\prime \prime }$ moduli, respectively associated with energy storage and dissipation during one deformation cycle. The registered stress values are fitted to the expression:

(10) $$\sigma_{\alpha \beta }\left(t\right)=\sigma_{0}\sin\left(\omega t+\delta \right).$$

The parameters extracted from the fit ($\sigma_{0}$ and δ) are used to calculate the real and imaginary components of the modulus:

(11) $$G^{\prime }=\frac{\sigma_{0}}{\gamma_{0}}\cos\delta ,\;G^{\prime \prime }=\frac{\sigma_{0}}{\gamma_{0}}\sin\delta .$$

They give information about the relaxation times characterising the material. A higher $G^{\prime }$ stands for predominant elastic component (typical of elastic solids), while a higher $G^{\prime \prime }$ stands for predominant viscous component (typical of simple liquids).

Rubber and polymer melts are ‘viscoelastic’, as both $G^{\prime }$ and $G^{\prime \prime }$ are are appreciably different from zero at typical testing frequencies. The response of unfilled rubbers is linear, as their moduli are almost independent of shear amplitude. Instead the Payne effect, which is normally associated with rubber reinforcement [10,12] but can be observed also in filled melts [77], involves a marked dependence of $G^{\prime }$ and $G^{\prime \prime }$ on the deformation amplitude. This typically occurs around 1% deformation. As mentioned in the Introduction, its origin is still controversial. The aim of the present simulation set-up is to analyse the dynamic and configurational properties of the confined chains under different but well-defined conditions, in order to extract molecular-level information about this phenomenon.

As discussed in the Methods section, we have performed MD simulations on the uncured, 100-mer polymer melt at T= 250 K. Other simulations have proved that cross-linking reduces the non-affine displacement and the segregation of NPs [35] and the Payne effect [78]. Although the cross-linking is not considered in this work, the connection between the surfaces is guaranteed for system $l_{1}$ and $l_{2}$, where there is an appreciable population of bridging chains. In fact, 90% of the chains in $l_{1}$ and 17% of those in $l_{2}$ have atoms within both surface layers. For the widest system ($l_{3}$), no chains are found to link the two surfaces.

**Figure 7:**
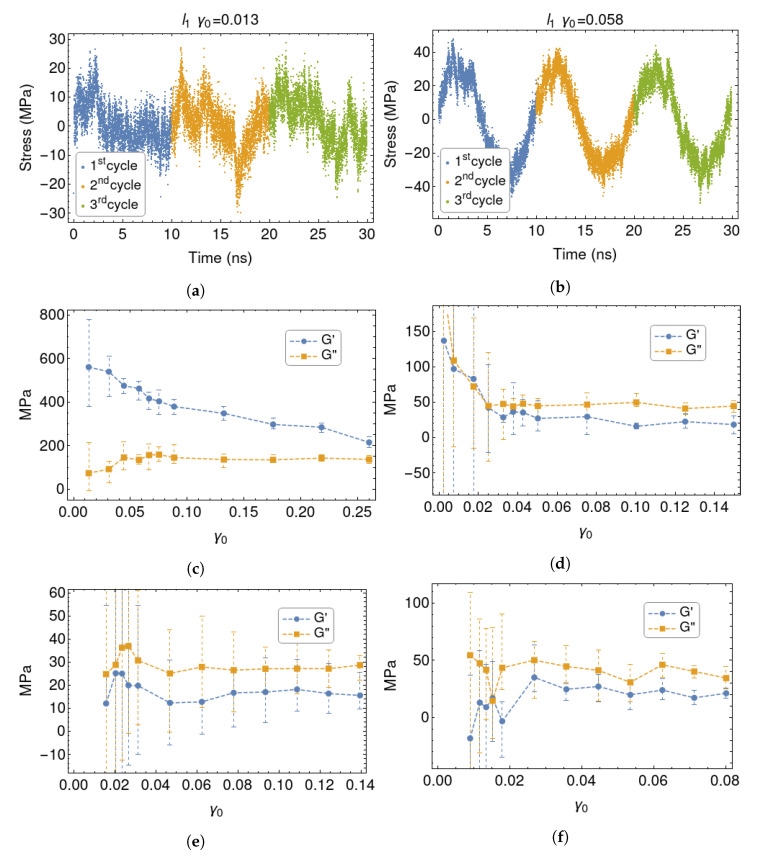
Stress registered in system *l*_1_ at low (**a**) and moderate (**b**) *γ*_0_ for three cycles. *G*′ and *G*′′ for systems $l_{1}$ (**c**), $l_{1}^{*}$ (**d**), $l_{2}$ (**e**) and $l_{3}$ (**f**).

The results of two representative DMA simulations are shown in Figure 7a,b. At small strains, the stress signal is partially masked by the noise, as it often happens in simulations of systems of limited dimension. Nonetheless, there are clearly different behaviours among the considered systems. Figure 7 shows the variation of the moduli with $\gamma_{0}$. The error bars reported on the plot are built using the maximum and minimum values extracted by fitting every complete deformation cycle. The first deformation cycle was not included in the fits, to exclude possible transient or “startup” effects. Both moduli change inversely with the inter–surface distance. Only system $l_{1}$ shows a solid-like behaviour, with $G^{\prime }>G^{\prime \prime }$. Also, their amplitude dependence resembles a typical experimental plot for the Payne effect. There is a large drop (>50%) in $G^{\prime }$, happening around $\gamma_{0}$ = 2.5%. Concurrently, there is also a rise and then a shallow drop in $G^{\prime \prime }$. This behaviour is not observable in systems $l_{2}$ and $l_{3}$, which are also characterised by strong noise at low $\gamma_{0}$. These systems have a prevalent liquid-like behaviour ($G^{\prime \prime }>G^{\prime }$), as expected since $T>T_{g}$. This allows us to conclude that the present simulations are relevant for the rubbery system, even if the deformation frequency is much larger than the one employed in typical experiments (but comparable to those employed in current non-equilibrium MD simulations). Also, the response of these systems reflects the dynamics of the underlying model for the polymer [54,55], and should be largely independent of the representation of the silica surfaces (atomistic or coarse-grained). Finally, in system $l_{1}^{*}$ we observe a drop in both $G^{\prime }$ and $G^{\prime \prime }$, which have a ratio close to unity at all amplitudes.

**Figure 8:**
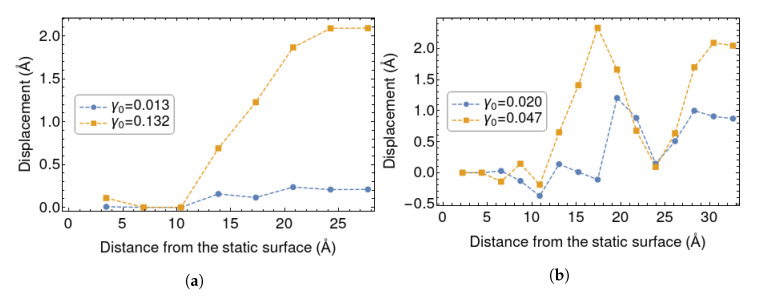
Shear displacement in the polymer layer for system $l_{1}$ (**a**) and $l_{2}$ (**b**) before and after the drop in *G*′ at the maximum *γ* reached during the DMA. On the *x* axis there is the distance from the static surface, on the y axis there is the average lateral displacement of the atoms belonging to a certain layer.

Figure 8 shows the average displacement field calculated inside the polymer layer for systems $l_{1}$ and $l_{2}$, at two strain amplitues. It is clear that in system $l_{1}$ the polymer follows the displacement more affinely. Most of the polymeric material within it can be considered to be “bound rubber”, and its mechanical response tends to support interpretations of the Payne effect associated with its non-liner mechanical properties. In system $l_{2}$ the atoms that lie in the center of the sandwich do not follow a simple, Couette-like linear profile. Indeed, the situation in $L_{2}$ is quite complex as there are at least three populations of chains: those which form bridges between the surfaces and deform more affinely, those which are not bonded to any surface and should be less affected, and those which are anchored to a single surface. Further simulations and analyses on larger systems should be carried out in order to fully clarify this issue.

## 5. Conclusions

We have built and tested a coarse-grained force field for silica in contact with a united-atom polybutadiene melt. From the outset, our aim was to develop a model retaining some chemical detail, but simple enough to allow large scale simulations of rubber nanocomposites based on these materials. The coarse-grained parameters for silica succeed in reproducing the structural properties of the polymer interacting with it, as measured by its density profiles next to two types of confining surfaces. The dynamical properties of the melt in contact with the coarse-grained silica models have also been tested and are generally consistent with the reference atomistic results, but with a margin of error. This is not surprising, as any coarse-graining procedure involves a loss of some chemical detail, and this tends to have a larger impact on the dynamical properties than on the structural ones [56,57,58].

We have also probed the viscoelastic properties of the sandwich model used to parametrize the silica force field. The system consisting of long polymer chains stuck between two closely spaced surfaces has a non-linear response to increasing shear amplitudes reminiscent of the Payne effect, as it is commonly observed in dynamic mechanical analyses of filled rubbers. This links the origin of the Payne effect to phenomena occurring within the polymer layer between particles, also known as bound rubber. In agreement with experiments, the effect is enhanced by increasing the filler concentration, which translates into shorter distances between the surfaces in our simulations.

The present model represents our first step towards the development of rubber–silica models incorporating a certain degree of chemical detail, unlike the generic bead-and-spring models which have been mostly used so far. In the future, we plan to apply it to more complex systems consisting of cross-linked polymer networks, incorporating actual nanoparticles with different concentrations and morphologies.

## Figures and Tables

**Table 1:** Non-bonded parameters for silica obtained from the Boltzmann inversion. Units for ϵ and σ are kcal/mol and Å respectively.

	CH${}_{2}$		CH		Q^3^		Q^4^	
	ϵ	σ	ϵ	σ	ϵ	σ	ϵ	σ
**CH${}_{2}$**	0.0936	4.500						
**CH**	0.1015	4.257	0.1000	3.800				
**Q^3^**	0.0967	5.065	0.1000	4.654	0.1000	5.700		
**Q^4^**	0.1676	4.450	0.1732	4.089	0.1732	5.008	0.3000	4.400

Note: Parameters for CH and CH${}_{2}$ are obtained from [55] and [54]. Cross interactions are obtained using geometric mixing rules, except for (CH, CH${}_{2}$).

**Table 2:** Activation energies related to the polymer diffusion in the *z* direction.

Silica Surface Model	Ea (kcal/mol)
AA $Q^{4}$	4.11
CG $Q^{4}$	3.27
AA $Q^{3}$	2.41
CG $Q^{3}$	3.41

**Table 3:** Parameters for the fit of the desorption function.

T (K)	Surface	τ (ps)	β	$\tau_{\mathrm{eff}}$ (ps)
325	AA Q${}^{4}$	2269	0.488	4751
	CG Q${}^{4}$	2994	0.497	6055
	AA Q${}^{3}$	2536	0.517	4777
	CG Q${}^{3}$	2736	0.506	5354
298	AA Q${}^{4}$	3457	0.520	6448
	CG Q${}^{4}$	2620	0.535	4660
	AA Q${}^{3}$	3446	0.590	5301
	CG Q${}^{3}$	3788	0.562	6242
273	AA Q${}^{4}$	5463	0.593	8348
	CG Q${}^{4}$	5490	0.562	9046
	AA Q${}^{3}$	6187	0.545	10,684
	CG Q${}^{3}$	5420	0.550	9227
250	AA Q${}^{4}$	7176	0.524	13,211
	CG Q${}^{4}$	7336	0.531	13,209
	AA Q${}^{3}$	12,037	0.705	15,134
	CG Q${}^{3}$	10,335	0.654	14,025
